# Time to sputum culture conversion and its associated factors among drug-resistant tuberculosis patients: a systematic review and meta-analysis

**DOI:** 10.1186/s12879-024-09009-5

**Published:** 2024-02-07

**Authors:** Yang Wenlu, Zhao Xia, Wu Chuntao, Yu Qiaolin, Xiao Xujue, Yao Rong, Su Dan, Yan Xi, Wan Bin

**Affiliations:** 1 Nursing Department, Public Health Clinical Center of Chengdu, Chengdu, Sichuan China; 2 Chengdu University of Traditional Chinese Medicine, Chengdu, Sichuan China

**Keywords:** DR-TB, Sputum culture conversion time, Risk factors, Meta-analysis

## Abstract

**Objective:**

We aimed to evaluate the sputum culture conversion time of DR-TB patients and its related factors.

**Methods:**

PubMed, The Cochrane Library, Embase, CINAHL, Web of Science, CNKI, Wan Fang, CBM and VIP databases were electronically searched to collect studies on sputum culture conversion time in patients with DR-TB. Meta-analysis was performed by using the R 4.3.0 version and Stata 16 software.

**Results:**

A total of 45 studies involving 17373 patients were included. Meta-analysis results showed that the pooled median time to sputum culture conversion was 68.57 days (IQR 61.01,76.12). The median time of sputum culture conversion in patients with drug-resistant tuberculosis was different in different WHO regions, countries with different levels of development and different treatment schemes. And female (*aHR* = 0.59,95%*CI: s*0.46,0.76), alcohol history (*aHR* = 0.70,95%*CI:*0.50,0.98), smoking history (a*HR* = 0.58,95%*CI:*0.38,0.88), history of SLD use (*aHR* = 0.64,95%*CI:*0.47,0.87), BMI < 18.5 kg/m^2^ (*aHR* = 0.69,95%*CI:*0.60,0.80), lung cavity (*aHR* = 0.70,95%*CI:*0.52,0.94), sputum smear grading at baseline (Positive) (*aHR* = 0.56,95%*CI:*0.36,0.87), (grade 1^+^) (*aHR* = 0.87,95%*CI:*0.77,0.99), (grade 2^+^) (*aHR* = 0.81,95%*CI:*0.69,0.95), (grade 3^+^) (*aHR* = 0.71,95%*CI:*0.61,0.84) were the related factor of sputum culture conversion time in patients with DR-TB.

**Conclusion:**

Patients with DR-TB in Europe or countries with high level of economic development have earlier sputum culture conversion, and the application of bedaquiline can make patients have shorter sputum culture conversion time. Female, alcohol history, smoking history, history of SLD use, BMI < 18.5 kg/m^2^, lung cavity, sputum smear grading at baseline (Positive, grade 1^+^, grade 2^+^, grade 3^+^) may be risk factors for longer sputum culture conversion time.

This systematic review has been registered in PROSPERO, the registration number is CRD42023438746.

**Supplementary Information:**

The online version contains supplementary material available at 10.1186/s12879-024-09009-5.

## Introduction

At present, the global situation of DR-TB (drug-resistant tuberculosis) is grim.

According to the 2022 Global Tuberculosis report, it is estimated that the number of new cases of MDR/RR-TB (multidrug-resistant/rifampin-resistant tuberculosis) reaches 450,000 [1]. Compared with drug-sensitive tuberculosis, drug-resistant tuberculosis, especially multidrug-resistant tuberculosis, has the characteristics of longer course of disease (18–24 months), heavy economic burden, more adverse reactions and poor therapeutic effect. A Meta analysis shows that the current success rate of MDR-TB treatment is only 58.4% [2], which is a major challenge in the field of tuberculosis treatment.

Because the treatment results of MDR-TB can not be obtained until 18–24 months after treatment, the effectiveness of the treatment regimen can not be evaluated in time, which to a certain extent affects the timely and effective adjustment of the treatment regimen. Therefore, the early prediction of treatment outcome of MDR-TB is very important [3]. The current evidence shows that sputum culture conversion time and status can be regarded as effective alternative indicators of treatment outcomes in patients with drug-resistant tuberculosis [4]. The study found that the earlier the sputum negative conversion, the better the efficacy [5], and faster conversion of sputum culture can increase patient comfort by reducing the duration of injectable drug use and simplifying patient treatment [6, 7]. In addition, the shorter sputum culture negative conversion time means that DR-TB patients have less chance of transmitting Mycobacterium tuberculosis, which has clinical and public health significance for controlling the spread of DR-TB [7]. Therefore, it is particularly important to evaluate the sputum culture conversion time and explore its influencing factors.

There are many studies on the sputum culture conversion time and its influencing factors in patients with DR-TB, but according to the current research results, it is found that there are differences in sputum culture conversion time among different studies, and the influencing factors are not consistent. At present, a systematic review describing the sputum culture conversion time and influencing factors in patients with MDR-TB is only for East African countries, and the inclusion of literature is limited, which limits the Meta analysis of influencing factors [8]. Therefore, we have the motivation to explore the sputum culture conversion time and related factors in the treatment of DR-TB patients. To further understand the level of treatment in each region and the differences between them. At the same time, to help medical staff identify the factors affecting the sputum culture conversion time, and intervene in time to improve the clinical outcome.

## Methods

### Search strategy

PubMed, The Cochrane Library, EMbase, CINAHL, Web of Science, CNKI, WanFang, CBM, VIP databases were electronically searched and retroactively included in the references of the study. The search time limit is from the establishment of the database to May 2023. Language restrictions are Chinese and English. During the search process, the authors used the following keywords and MeSH terms: “Drug-Resistant Tuberculosis/DR-TB/MDR-TB/Multidrug-Resistant Tuberculosis/MDR Tuberculosis/Extensively drug resistant pulmonary tuberculosis/XDR-TB/RR-TB” and “Sputum culture conversion time/Sputum conversion”.

### Selection criteria

Inclusion criteria: On the one hand, the subjects were clearly diagnosed as DR-TB patients, and the content of the study reported the median time of sputum culture conversion (Median time, IQR) during the treatment of DR-TB patients, on the other hand, the type of study was a cohort study. Sputum culture conversion is defined as two consecutive negative sputum cultures at an interval of at least one month (or four weeks) after the initial positive sputum culture. The negative conversion time of sputum bacteria was the collection time of sputum culture negative samples for the first time [9].

Exclusion criteria: (1) reviews or case reports; (2) duplicate studies; (3) original texts were not in English or Chinese; (4) data were incomplete; (5) the full text can not be obtained.

### Study selection

References were stored and managed using Endnote X9. The articles retrieved from the databases were imported to Endnote X9, and then duplicates were removed. Two researchers independently conducted the screening of the research literature. Articles were first screened based on the title and abstract, and then the literature was re-screened by reading the full text. In case of disagreement between the two researchers, a third researcher was consulted.

### Data extraction and quality assessment

Data extraction using a standardized Microsoft Excel data extraction tool was carried out by two independent authors for each study, and inconsistencies were resolved by consultation with a third author. The contents of data extraction included the first author of the literature, year of publication, country, region according to WHO, data year, type of study, sample size, average/median age of patients, median time of sputum culture (IQR), 2-month negative conversion rate, overall negative conversion rate, treatment scheme, influencing factors of negative conversion time. Newcastle–Ottawa scale (Newcastle–Ottawa Scale, NOS) was used to evaluate the quality of the literature [10].

### Data processing and analysis

Meta-analysis of sputum culture conversion time of DR-TB patients was performed with R software version 4.3.0 and the combination of the median time (median, IQR) to sputum culture conversion was realized by QE method (Quantile estimation, QE); Meta-analysis of the influencing factors of sputum culture conversion time was performed with software version 16 and the effect was combined with hazard ratio (*HR*) and its 95% confidence interval (*CI*). Heterogeneity was assessed by computing p-values of Higgins’s *I*^*2*^test statistics and Q-statistics among reported median time of culture conversion. If *P* > 0.1 and *I*^*2*^ < 50%, it shows that there is no statistical heterogeneity among the studies, so choose the fixed effect model, otherwise suggest that there is statistical heterogeneity, and choose the random effect model. The Higgins’s* I*^*2*^ statistic measures the difference between sample quartile estimation, which is due to heterogeneity due to random error rather than to sampling error. In this case, the pooled effect was estimated with a random-effects meta-analysis model. Subgroup analyses were performed to identify possible sources of heterogeneity by considering WHO region the study belonged to, treatment regimens, and national development level. The heterogeneity of the results was analyzed by χ^2^ test (the test level was α = 0.1) when the factors affecting the sputum culture conversion time were analyzed. If there was no statistical heterogeneity among the results (*p* > 0.1, *I*^*2*^ < 50%), the fixed-effects model was used for Meta-analysis. If there was statistical heterogeneity among the results (*p* ≤ 0.1, *I*^*2*^ ≥ 50%), random effects model was used for Meta-analysis. The test level of Meta analysis was 0. 05. The publication bias was analyzed by funnel chart.

## Results

**Fig. 1:**
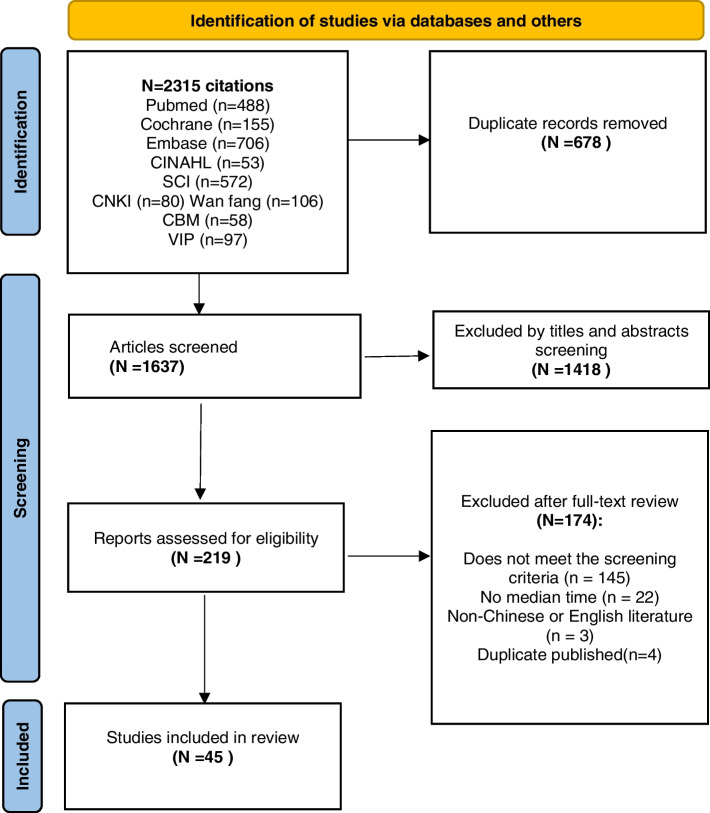
Flow chart of selecting articles for systematic review and meta-analysis

A total of 2315 articles were retrieved, and 45 studies including 17,373 DR-TB patients were finally included after layer-by-layer screening. The flow chart and results of literature screening are shown in Fig. 1.

### Characteristics of the included studies

The 45 articles included were published from 2011 to 2022, from Ethiopia, Nigeria, Indonesia, China, Georgia, South Africa, Tanzania, Kenya, Egypt, Peru, South Korea, Pakistan, Botswana, Nepal, Liwan Tao, India, Guinea, Germany, Myanmar, Dominica 20 countries. Distributed in Africa, Southeast Asia, Western Pacific, Europe, America, Eastern Mediterranean six WHO regions. All were cohort studies, with data from 1990 to 2020 and study sample sizes ranging from 16 to 3712 with a cumulative total of 17,373 patients. The basic characteristics of the included literatures are shown in Table 1. (As the width of the table exceeds the letter landscape page, please see the Additional files).

**Table 1:** Descriptive summary of included studies

Authors	Year (yr)	Country	Region by World Health Organization	Year of the data (yr)	Study design	Sample size	Mean/median, age (yrs)	Type of TB	Median time (IQR)	2nd-month Conversion (%)	Overall conversion (%)	Treatment scheme	Influencing factors of conversion time
Akalu TY	2018	Ethiopia	Africa	2010–2016	Retrospective cohort	392	29.5	RR-TB MDR-TB XDR-TB	65 days (60–70)	-	86.7%	-	3, 8, 12, 13, 14
Akinsola OJ	2018	Nigeria	Africa	2012–2016	Retrospective cohort	413	36.8 ± 12.7	MDR-TB	5.5 months (1.5–11.5)	-	58.4%	-	1, 6, 15
Putri FA	2014	Indonesia	Southeast Asia	2009–2011	Retrospective cohort	212	37 ± 12	MDR-TB XDR-TB	2 months (1–3)	-	81%	A + B	2, 5, 8, 14, 17, 19
Lu P	2017	China	Western Pacific region	2011–2014	Prospective cohort	139	51	MDR-TB	92 days (34–111)	28%	76.3%	A + B	-
Li Q	2019	China	Western Pacific region	2011–2015	Retrospective cohort	365	-	MDR-TB	85 days (42.0–106.5)	-	90.96%	A + B	-
Kurbatova EV	2012	Multiple countries	Multi-region	2000–2003	Retrospective cohort	1416	38	MDR-TB XDR-TB	3 months (2.0–5.0)	33%	85.4%	-	-
Li Q	2020	China	Western Pacific region	2011–2015	Retrospective cohort	384	41.7 ± 15.4	MDR-TB	85 days (40–112)	-	93.5%	-	1
Liu Q	2018	China	Western Pacific region	2011–2014	Prospective cohort	139	51	MDR-TB	91.5 days (34.0–110.8)	-	76.3%	A + B	1, 2, 3, 4, 8,19
Magee MJ	2014	Georgia	Europe	2009–2011	Prospective cohort	1366	35.1	MDR-TB	68 days (50–120)	-	70.7%	B	1, 2, 3, 4, 5, 6, 8, 10, 12, 18
Ncha R	2019	South Africa	Africa	2012–2014	Retrospective cohort	371	38	MDR-TB XDR-TB	58.2 days (29–113)	-	70%	-	2, 3, 4, 5, 8, 12, 14
Mpagama SG	2013	Tanzania	Africa	2009–2011	Retrospective cohort	61	36 ± 13	MDR-TB	2 months (1–3)	-	85%	A	-
Huerga H	2017	Kenya	Africa	2006–2012	Retrospective cohort	169	29	MDR-TB	2 months (2–3)	-	73.9%	A + B	-
Bade AB	2021	Ethiopia	Africa	2013–2019	Retrospective cohort	200	32.9 ± 9.5	MDR-TB	31 days (30–61)	79.5%	100%	-	-
Brust JC	2011	South Africa	Africa	2008–2009	Retrospective cohort	45	33	MDR-TB	62 days (48–111)	-	89%	A	-
Magee MJ	2017	South Africa	Africa	2011–2015	Prospective cohort	91	35	MDR-TB	82 days (53–143)	55.5%	96%	A	-
Brust JC	2013	South Africa	Africa	2008–2010	Retrospective cohort	56	36	MDR-TB	66 days (43–96)	32%	95%	A	-
Gadallah MA	2016	Egypt	Africa	2006–2010	Prospective cohort	228	37	MDR-TB	60 days (58–121)	21.3%	87.5%	B	-
Zheng X	2022	China	Western Pacific region	2016–2019	Prospective cohort	197	42.0 ± 9.9	MDR-TB	4 months (2–14)	44.7%	79.2%	A	-
Tierney DB	2014	Peru	America	1990–2002	Retrospective cohort	592	28.7	MDR-TB	59 days (31–92)	-	87.7%	B	-
Lee M	2019	South Korea	Southeast Asia	2011–2015	Retrospective cohort	2472	47 ± 17.0	MDR-TB	61 days (28–109)	-	-	B	-
Javaid A	2018	Pakistan	Eastern Mediterranean	2012–2014	Retrospective cohort	428	30.7 ± 14.35	MDR-TB	58 days (30–90)	56.8%	-	B	8, 11, 12, 15, 16, 19
Hafkin J	2013	Botswana	Africa	2005–2009	Retrospective cohort	HIV-infected:40;non-HIV-infected:30	38	MDR-TB	HIV-infected:78 days (42–186) non-HIV-infected:95 days (70–133)	-	84%	B	7
Ghimire S	2020	Nepal	Southeast Asia	2014–2016	Retrospective cohort	98	29	MDR-TB	60 days (60–90)	-	82.6%	A	-
Diktanas S	2021	Li Wantao	Europe	2016–2019	Retrospective cohort	115	48 ± 12	MDR-TB RR-TB XDR-TB	1.1 months (0.9–1.8)	65.2%	89.6%	-	1, 2, 3, 4, 8, 11, 12, 14
Meshesha MD	2022	Ethiopia	Africa	2014–2018	Retrospective cohort	145	29.6 ± 12.4	MDR/RR-TB	2 months (1–3)	69.7%	-	A + B	2, 9, 10, 12, 14
Tekalegn Y	2020	Ethiopia	Africa	2012–2017	Retrospective cohort	228	28	All DR-TB	61 days (34–92)	46.9%	-	A	5, 12, 14
Velayutham B	2016	India	Southeast Asia	2009–2011	Retrospective cohort	787	-	MDR/RR-TB	91.3 days (91.3–121.7)	-	83%	A	-
Diallo A	2020	Guinea	Africa	2016–2018	Retrospective cohort	118	34	RR-TB	59 days (31–61)	-	89%	A	-
Reimann M	2019	Germany	Europe	2012–2017	Retrospective cohort	non-smoker:20; smoker:45	36.5/37.4	MDR-TB XDR-TB	non-smoker:53 days (19–89) smoker:60.7 days (33.3–76)	-	-	-	-
Shibabaw A	2018	Ethiopia	Africa	2011–2016	Retrospective cohort	235	30	MDR/RR-TB	72 days (44–123)	-	85.5%	A	5, 7
Ding CH	2021	China	Western Pacific region	2018–2020	Prospective cohort	79	≥ 18	MDR-TB XDR-TB pre-XDR-TB	4 weeks (2–8)	70.9%	91.1%	C	-
Htun YM	2018	Myanmar	Southeast Asia	2014	Retrospective cohort	330	39.45 ± 13.28	MDR-TB	147 days (94–241)	-	-	A	-
Shi ZY	2021	China	Western Pacific region	2018–2019	Retrospective cohort	38	31	MDR-TB/ XDR-TB	8 weeks (4–16)	68.4%	84.2%	C	-
Kim CT	2018	South Korea	Southeast Asia	2015–2017	Retrospective cohort	55	52	MDR-TB XDR-TB	119 days (52.5–198.5)	-	70.9%	C	-
Abubakar M	2022	Pakistan	Eastern Mediterranean	2010–2017	Retrospective cohort	355	32.99 ± 14.54	XDR-TB	91 days (59–156)	27.3%	63.6%	B	1, 8, 10, 15, 16, 20
Salindri AD	2016	Georgia	Europe	2011–2014	Prospective cohort	52	47	MDR-TB XDR-TB	62 days (32–94)	-	84.6%	-	1, 2, 4, 6, 7, 8, 12
Wu GL	2021	China	Western Pacific region	2018–2020	Prospective cohort	16	26 ~ 27	MDR-TB XDR-TB	8 weeks (4–12)	-	94%	C	-
Pei Y	2021	China	Western Pacific region	2018–2020	Prospective cohort	44	38	MDR-TB XDR-TB	22 days (18–59)	-	95%	C	-
Kim J	2016	South Korea	Southeast Asia	2009–2012	Retrospective cohort	35	41	MDR-TB XDR-TB	56 days (0–92)	60%	-	A	-
Shi L	2021	China	Western Pacific region	2018–2019	Retrospective cohort	DM group: 76; non-DM group: 61	49.8 ± 10.5 49.3 ± 9.7	MDR-TB XDR-TB	56 days (28–63) 56 days (28–84)	-	95.6% 98.2%	C	-
Rodriguez M	2013	Dominica	America	2006–2010	Retrospective cohort	289	35	MDR-TB	2 months (2–3)	-	86.5%	A + B	-
Parmar MM	2018	India	Southeast Asia	2007–2011	Retrospective cohort	3712	35	MDR-TB	100 days (92–125)	-	73.6%	-	1, 2, 5, 6
Heyckendorf J	2018	Germany	Europe	2013–2016	Prospective cohort	29	36	MDR-TB XDR-TB	39 days (6–85)	61%	95%	B + C	-
Gao M	2021	China	Western Pacific region	2018.02–12	Prospective cohort	177	40	MDR-TB XDR-TB	4 weeks (2–8)	67.2%	85.3%	C	-
Borisov SE	2017	Multiple countries	Multi-region	2008–2016	Retrospective cohort	428	35	MDR-TB XDR-TB	60 days (33–90)	56.7%	91.2%	C	-

A = a standardized regimen; B = an individualized regimen; C = bedaquiline-containing regimens

Note:- = Not described; 1 = Age; 2 = Sex; 3 = Alcohol; 4 = Smoking status; 5 = BMI, kg/m^2^; 6 = Diabetes; 7 = HIV status; 8 = Sputum smear grading at baseline; 9 = Baseline Hemoglobin (g/dl); 10 = TB treatment history; 11 = History of SLD use; 12 = lung cavity; 13 = Consolidation; 14 = Type of resistance; 15 = Number of resistant drugs; 16 = Resistance to all five first lines drugs; 17 = Resistant to any injectable(s); 18 = Any 2nd line resistance; 19 = Resistance to ofloxacin; 20 = Use of high dose isoniazid

### Literature quality evaluation

All the included studies were evaluated strictly according to NOS standards, with 13 of medium quality and 32 of high quality (Table 2).

**Table 2:** Results of bias risk assessment (Score)

	Name	Selection				Comparability	Outcome			Score	Quality Grade
		①	②	③	④	⑤	⑥	⑦	⑧		
1	Akalu TY	1	1	1	0	2	1	1	1	8	High quality
2	Akinsola OJ	1	1	1	0	2	1	1	1	8	High quality
3	Putri FA	1	1	1	0	2	0	1	1	7	Moderate quality
4	Lu P	1	1	1	1	2	1	1	1	9	High quality
5	Li Q	1	1	1	0	2	1	1	1	8	High quality
6	Kurbatova EV	1	1	1	0	2	1	1	1	8	High quality
7	Li Q	1	1	1	0	2	1	1	1	8	High quality
8	Liu Q	1	1	1	1	2	1	1	1	9	High quality
9	Magee MJ	1	1	1	1	2	1	1	1	9	High quality
10	Ncha R	1	1	1	0	2	1	1	1	8	High quality
11	Mpagama SG	1	1	1	0	2	0	1	1	7	Moderate quality
12	Huerga H	1	1	1	0	2	1	1	1	8	High quality
13	Bade AB	1	1	1	1	2	0	1	1	8	High quality
14	Brust JC	1	1	1	0	1	1	1	1	7	Moderate quality
15	Magee MJ	1	1	1	1	2	1	1	1	9	High quality
16	Brust JC	1	1	1	0	2	1	1	1	8	High quality
17	Gadallah MA	1	1	1	1	2	1	1	1	9	High quality
18	Zheng X	1	1	1	1	2	1	1	1	9	High quality
19	Tierney DB	1	1	1	0	2	0	1	1	7	Moderate quality
20	Lee M	1	1	1	0	2	1	0	1	7	Moderate quality
21	Javaid A	1	1	1	0	2	1	1	1	8	High quality
22	Hafkin J	1	1	1	0	2	0	0	1	6	Moderate quality
23	Ghimire S	1	1	1	0	2	1	1	1	8	High quality
24	Diktanas S	1	1	1	0	2	1	1	1	8	High quality
25	Meshesha MD	1	1	1	0	2	0	0	1	6	Moderate quality
26	Tekalegn Y	1	1	1	0	2	1	1	1	8	High quality
27	Velayutham B	1	1	1	0	2	1	1	1	8	High quality
28	Diallo A	1	1	1	0	2	1	1	1	8	High quality
29	Reimann M	1	1	1	0	2	1	0	1	7	Moderate quality
30	Shibabaw A	1	1	1	0	2	1	0	1	7	Moderate quality
31	Ding CH	1	1	1	1	2	1	1	1	9	High quality
32	Htun YM	1	1	1	0	2	1	1	1	8	High quality
33	Shi ZY	1	1	1	1	2	1	1	1	9	High quality
34	Kim CT	1	1	1	0	1	1	0	1	6	Moderate quality
35	Abubakar M	1	1	1	0	2	1	0	1	7	Moderate quality
36	Salindri AD	1	1	1	1	2	1	1	1	9	High quality
37	Wu GL	1	1	1	1	0	1	1	1	8	High quality
38	Pei Y	1	1	1	1	1	1	1	1	8	High quality
39	Kim J	1	1	1	0	2	1	0	1	7	Moderate quality
40	Shi L	1	1	1	0	2	1	1	1	8	High quality
41	Rodriguez M	1	1	1	0	2	1	1	1	8	High quality
42	Parmar MM	1	1	1	0	2	1	1	1	7	Moderate quality
43	Heyckendorf J	1	1	1	1	2	1	1	1	9	High quality
44	Gao M	1	1	1	1	2	1	1	1	9	High quality
45	Borisov SE	1	1	1	0	2	1	1	1	8	High quality

Note: ①Representativeness of the exposed cohort; ②selection of the non exposed cohort; ③Ascertainment of exposure; ④Demonstration that outcome of interest was not present at start of study; ⑤Comparability of cohorts on the basis of the design or analysis; ⑥Assessment of outcome; ⑦Was follow-up long enough for outcomes to occur; ⑧Adequacy of follow up of cohorts

### Time to sputum culture conversion among DR-TB patients

The median time of sputum culture conversion was described in all 45 studies. Meta analysis showed that the pooled median time of sputum culture conversion was 68.57d (IQR 61.01,76.12). According to the Higgins *I*^*2*^ test (*I*^*2*^ = 99.32%, *p* < 0.0001), the pooled median time of sputum culture conversion in Meta analysis showed high heterogeneity. Therefore, we conducted a subgroup analysis to determine the source of heterogeneity. We considered the subgroup analysis of the characteristics of the inclusion study, such as WHO region, national development level, treatment scheme and so on. By region, Meta analysis showed that the shortest negative conversion time of sputum culture was 53.15 days (IQR 40.39,65.91) in Europe, and the longest negative conversion time of sputum culture was 85.94 days (IQR 63.00,108.88) in Southeast Asia. According to the national development level, Meta analysis shows that the negative conversion time of developed countries is 57.63 days (IQR 40.48,74.78), while that of developing countries is 69.97 days (IQR 61.35,78.59). According to the analysis of whether the treatment regimen contained bedaquiline or not, the results of Meta analysis showed that sputum culture conversion time was 49.39 days (IQR 34.95,63.83) in patients with bedaquiline and 73.36 days (IQR 65.68,81.04) in patients without bedaquiline in treatment regimen (Table 3).

**Table 3:** Subgroup analysis of sputum culture conversion time among DR-TB patients

Method	Pooled median time (IQR), d		Heterogeneity evaluation		
			*T*^2^	*I*^*2*^	Q
QE	68.57 (61.01, 76.12) d		663.34	99.32%	5149.79 (*P* < 0.0001)
Category	Subgroup	No. of studie	Sample size	Median time (IQR),d	*P*-val
World Health Organization regions	Africa	15	2780	69.42d (56.35, 82.49)	< 0.0001 (*I*^*2*^ = 98.71%)
	Europe	5	1627	53.15d (40.39, 65.91)	< 0.0001 (*I*^*2*^ = 92.37%)
	Southeast Asia	8	7701	85.94d (63.00, 108.88)	< 0.0001 (*I*^*2*^ = 99.73%)
	America	2	881	59.64d (56.92, 62.37)	0.73 (*I*^*2*^ = 0%)
	Eastern Mediterranean	2	787	59.22d (54.56, 63.88)	0.86 (*I*^2^ = 0%)
	Western Pacific Ocean	11	1715	63.27d (46.78, 79.76)	< 0.0001 (*I*^*2*^ = 97.39%)
National development level	developed country	6	2771	57.63d (40.48, 74.78)	< 0.0001 (*I*^2^ = 96.12%)
	developing country	37	12,716	69.97d (61.35, 78.59)	< 0.0001 (*I*^2^ = 99.41%)
Treatment regimen	without bedaquiline	26	8895	73.36d (65.68, 81.04)	< 0.0001 (*I*^2^ = 97.76%)
	Contain bedaquiline	9	1003	49.39d (34.95, 63.83)	< 0.0001 (*I*^*2*^ = 96.67%)

### Influencing factors of sputum culture conversion time

Among the 45 studies included in this study, 16 reported the adjusted *HR* values of the factors affecting the sputum culture conversion time, which were included in Meta analysis. Finally, 12 influencing factors were included in the analysis, including gender, alcohol, smoking status, TB treatment history, history of second-line drug (SLD) use, BMI, diabetes, lung cavity, HIV, sputum smear grading at baseline (Positive, grade 1^+^, grade 2^+^, grade 3^+^), resistance to ofloxacin, and resistance to all five first lines drugs.

The results of Meta analysis showed that female, alcohol history, smoking history, history of SLD use, BMI < 18.5 kg/m^2^, lung cavity and sputum smear grading at baseline (Positive, grade 1^+^, grade 2^+^, grade 3^+^) were the influencing factors of longer sputum culture conversion time, and fund to be statistically significant (*P* < 0.05). However, male, current smoking, TB treatment history, diabetes, HIV, resistance to ofloxacin, and resistance to all five first lines drugs were not the influencing factors of longer sputum culture conversion time (Table 4).

**Table 4:** Meta analysis of the factors affecting the sputum culture conversion time among DR-TB patients

The influence factors were included	Exposure factors	Number of articles included	Heterogeneity			*aHR*	95%*CI*	*P*
			*I*^*2*^ (%)	*P*	effects model			
Sex	Male	4	0	0.57	Fixed	0.99	0.91 ~ 1.07	0.80
	Female	3	0	0.84	Fixed	0.59	0.46 ~ 0.76	< 0.0001
Alcohol	Alcohol history	3	49	0.14	Fixed	0.70	0.50 ~ 0.98	0.039
Smoking status	Smoking history	3	0	0.506	Fixed	0.58	0.38 ~ 0.88	0.01
	Current smoker	2	79.2	0.028	Random	0.61	0.30 ~ 1.24	0.17
TB treatment history		3	0	0.925	Fixed	0.94	0.83 ~ 1.09	0.46
History of SLD use		2	0	0.96	Fixed	0.64	0.47 ~ 0.87	0.004
BMI	BMI < 18.5 kg/m^2^	4	0	0.89	Fixed	0.69	0.60 ~ 0.80	< 0.0001
Diabetes		4	79.1	0.002	Random	0.77	0.50 ~ 1.17	0.22
lung cavity		5	70.3	0.009	Random	0.70	0.52 ~ 0.94	0.016
HIV		2	0	0.673	Fixed	0.76	0.42 ~ 1.21	0.36
Sputum smear grading	Positive	3	50.3	0.13	Random	0.56	0.36 ~ 0.87	0.009
	grade 1 +	3	0	0.94	Fixed	0.87	0.77 ~ 0.99	0.043
	grade 2 +	3	0	0.66	Fixed	0.81	0.69 ~ 0.95	0.009
	grade 3 +	3	0	0.94	Fixed	0.71	0.61 ~ 0.84	< 0.0001
Resistance to ofloxacin		3	58.6	0.09	Random	0.67	0.43 ~ 1.04	0.07
Resistance to all five first lines drugs		2	0	0.973	Fixed	0.86	0.62 ~ 1.21	0.395

In addition, age, type of resistance, number of resistant drugs, consolidation, resistant to any injectable(s), resistance to any second-line drug, baseline hemoglobin (g/dl) and use of high-dose isoniazid could not be Meta-analyzed, because the classification criteria are different or only mentioned in a single article.

### Sensitivity analysis

In order to test the stability and reliability of the analysis results, the fixed effect model and random effect model were used to calculate *HR* and 95%*CI* respectively, and the stability of the results was discussed. The results showed that except for “Alcohol history”, “Current smoker” and “Resistance to ofloxacin”, the Meta analysis results of other risk factors did not change after the transformation effect model, which suggested that the results were reliable (Table 5).

**Table 5:** The combined results of fixed effect model and random effect model

The influence factors were included	Exposure factors	Fixed effects model	Random effect model
Gender	Male	0.99 (0.91 ~ 1.07)	0.99 (0.91 ~ 1.07)
	Female	0.59 (0.46 ~ 0.76)	0.59 (0.46 ~ 0.76)
Alcohol	Alcohol history	0.70 (0.50 ~ 0.98)	0.69 (0.43 ~ 1.13)
Smoking status	Smoking history	0.58 (0.38 ~ 0.88)	0.58 (0.38 ~ 0.88)
	Current smoker	0.79 (0.68 ~ 0.91)	0.61 (0.30 ~ 1.24)
TB treatment history		0.94 (0.83 ~ 1.09)	0.94 (0.83 ~ 1.09)
History of SLD use		0.64 (0.47 ~ 0.87)	0.64 (0.47 ~ 0.87)
BMI	BMI < 18.5 kg/m^2^	0.69 (0.60 ~ 0.80)	0.69 (0.60 ~ 0.80)
Diabetes		0.77 (0.50 ~ 1.17)	0.77 (0.50 ~ 1.17)
lung cavity		0.70 (0.62 ~ 0.80)	0.70 (0.52 ~ 0.94)
HIV		0.76 (0.42 ~ 1.21)	0.76 (0.42 ~ 1.21)
Sputum smear grading	Positive	0.58 (0.45 ~ 0.76)	0.56 (0.36 ~ 0.87)
	grade 1 +	0.87 (0.77 ~ 0.99)	0.87 (0.77 ~ 0.99)
	grade 2 +	0.81 (0.69 ~ 0.95)	0.81 (0.69 ~ 0.95)
	grade 3 +	0.71 (0.61 ~ 0.84)	0.71 (0.61 ~ 0.84)
Resistance to ofloxacin		0.66 (0.53 ~ 0.80)	0.67 (0.43 ~ 1.04)
Resistance to all five first lines drugs		0.86 (0.62 ~ 1.21)	0.86 (0.62 ~ 1.21)

### Publication bias

We did not assess publication bias due to the limited number of studies (< 10) [11].

## Discussion

This study comprehensively searched the study on the sputum culture conversion time in DR-TB, and finally included 45 articles that met the inclusion criteria. The included literatures come from 20 countries and are widely distributed in 6 WHO regions, with a total of 17,373 samples. All the literatures are cohort studies with strong causal argumentation intensity. The overall NOS quality scores included in the literature are all ≥ 6, indicating that the quality of literature methodology is medium or above, so the overall conclusion of the study is more reliable.

This study was divided into subgroups according to the characteristics of the literature, and discussed the sputum culture conversion time under different WHO regions, national development levels and treatment schemes, and used the adjusted HR value to analyze the influencing factors of sputum culture conversion time, so as to ensure the scientificity and reliability of the results. At the same time, this study is of great significance to explore the sputum culture conversion time and its influencing factors which are of great value in monitoring treatment results, preventing and controlling infection and adjusting patients’ treatment plan. Therefore, this study mainly focuses on the median time of sputum culture conversion in the treatment of DR-TB patients, and objectively analyzes the influencing factors of conversion time, in order to provide clinical reference.

The results of this study show that women have a longer conversion time of sputum culture than men. This difference may reflect the biological differences in patients with DR-TB.

Studies have shown that alcohol use is a key driver of poor response to tuberculosis treatment [12]. The results of this study showed that alcohol history was a risk factor for longer sputum culture negative conversion time in patients with DR-TB, which was consistent with the conclusions of previous studies. This may be due to the fact that alcohol can reduce the number and function of dendritic cells and neutrophils by inhibiting the phagocytic and bactericidal activity of macrophages, thus reducing the immune function of patients with DR-TB. In addition, some studies have pointed out that long-term heavy drinking is related to the inhibition of phagocytosis and the production of growth factors in innate immune cells in a dose-and time-dependent manner [13], indicating that long-term alcohol consumption has a greater adverse effect on the immune response of tuberculosis. Whether the length of drinking history and the severity of alcohol consumption further promote the delay of negative conversion time of sputum culture is still worthy of further exploration.

This study found that patients with a history of smoking had a longer negative conversion time of sputum culture than patients without a history of smoking. Published studies have shown that smoking can delay sputum culture transformation in tuberculosis patients, including XDR-TB [14], which is consistent with the findings of previous studies. It may be because smoking has a negative effect on the phagocytosis of alveolar macrophages, which leads to the spread of tuberculosis bacteria in the lungs and delays the clearance of bacteria [15].

The history of the use of second-line anti-tuberculosis drugs is the influencing factor of the sputum culture conversion time, which may be due to the more complex drug resistance caused by the exposure of patients to second-line anti-tuberculosis drugs, resulting in poor therapeutic effect and longer sputum culture conversion time [16]. BATOOL et al. also support this view [17]. In addition, BATOOL et al. also pointed out that the sputum culture conversion time of patients increased with the increase of previous exposure to SLD [17]. However, this study has not been explored because of the lack of relevant data in the literature, so it needs to be further studied.

The results of this study showed that malnutrition was a risk factor for longer sputum culture conversion time. A Meta analysis of the effect of malnutrition on sputum culture negative conversion time showed that malnutrition was significantly associated with longer sputum culture negative conversion time [18]. It is consistent with the conclusion of this study. In addition, some studies have found that obese patients with tuberculosis have a lower conversion rate than patients with ideal body mass index [19], so overweight and obesity may also delay the sputum culture conversion time, but this study only analyzed the effect of BMI < 18.5 kg/m^2^ on the sputum culture conversion time. The relationship between overweight, obesity and sputum culture negative conversion time needs to be further explored.

In this study, it was determined that lung cavity was a factor affecting the negative conversion time of sputum culture. On the one hand, it may be due to the high load of mycobacteria in patients with lung cavity [20]. On the one hand, it may be difficult for drugs to penetrate into these lung cavities. Reduce the drug permeability and antibacterial activity, and finally prolong the sputum conversion time [21]. In addition, the study [22] found that the median time of sputum culture transformation in patients with single lung cavity was shorter than that in patients with double lung cavity, but Tekaleg et al. found that the negative conversion time of sputum culture in patients with single lung cavity and double lung cavity was not statistically significant [22], so the relationship between the two needs to be further determined.

Patients with negative sputum smear at baseline took longer to turn negative than patients with sputum smear positive at baseline and sputum smear grades 1^+^, 2^+^ and 3^+^. It may be because of the high bacterial load, it takes a long time to remove the bacteria.

In this study, we have not found the correlation between the negative conversion time of sputum culture and the history of TB treatment, diabetes, HIV, resistance to ofloxacin and resistance to five first-line antituberculosis drugs. With regard to the history of TB treatment, most studies have found that retreated pulmonary tuberculosis patients have more bacterial load and later sputum bacteria conversion than newly treated pulmonary tuberculosis patients [23]. BADE et al. pointed out that patients with a previous history of TB treatment had a 4-fold higher risk of delayed culture conversion than patients with new MDR-TB [24]. It is not consistent with the conclusion of this study, which may be related to the lack of literature included in this study. Therefore, the relationship between the history of TB treatment and the negative conversion time of sputum culture needs further study. Previous studies showed that diabetes delayed the sputum culture conversion time of drug-sensitive tuberculosis [21], but this study showed that diabetes had no effect on the sputum culture negative conversion time of DR-TB. JAFRI et al. also indicated that the blood glucose level did not affect the sputum culture negative conversion rate of DR-TB patients when adopting the best regimen [23]. It is consistent with the conclusion of this study.

In addition, this study found that age, type of drug resistance, number of resistant drugs, consolidation, resistant to any injectable(s), resistance to any second-line drug, baseline hemoglobin (g/dl) and use of high dose isoniazid may be related to the negative conversion time of sputum culture in patients with DR-TB, but we were not able to pool to generate the effect size of these factors on sputum culture conversion time due to the different classification criteria or only mentioned in single study, so more studies are needed to confirm this furtherly.

Our limitations include: (1) Only Chinese and English literatures are included in this study, and there may be some selection bias; (2) The description of the median time of sputum culture conversion is not all in days. In this study, the conversion time in monthly /weekly units is converted into days, which may have some errors; (3) The description of the treatment schemes is not specific enough to further analyze its effect on the median time of negative conversion; (4) Some of the influencing factors can not be analyzed by Meta because of different classification criteria or only mentioned in a single article; (5) Since the number of studies included in the Meta analysis is less than 10, the funnel chart is not depicted, and there may be a potential publication bias. We found that in some studies, the monitoring frequency of sputum culture is not strictly once a month, which may affect the accuracy of sputum culture negative conversion time. Therefore, it is suggested that more prospective studies with high quality and large sample size be carried out in the future, strict monthly sputum examination, and further clarify the factors affecting the negative conversion time of sputum culture.

## Conclusion

Therefore, the negative effects of female, alcohol history, smoking history, history of SLD use, BMI < 18.5 kg/m^2^, lung cavity and sputum smear grading at baseline (Positive, grade 1^+^, grade 2^+^, grade 3^+^) on sputum negative conversion time should be recognized. Patients with this characteristic should be prevented and reliable intervention programs should be adjusted to improve the prognosis of patients.

### Supplementary Information

**Additional file 1.** 

**Additional file 2.** 

## Data Availability

The dataset(s) supporting the conclusions of this article is (are) included within the article (and its additional file(s)).
