# Supplementary material for: Time to sputum culture conversion and its associated factors among drug-resistant tuberculosis patients: a systematic review and meta-analysis
Source: BMC Infect Dis. 2024 Feb 7;24:169. doi: 10.1186/s12879-024-09009-5 (PMC10848338; doi:10.1186/s12879-024-09009-5)
Supplement: Supplementary file 1 — Additional file 1. [file 12879_2024_9009_MOESM1_ESM.docx]

**Forest plot performed by stata (Analysis of related factors of sputum culture conversion time)**

1. Gender (male as the exposure factor) ：[*aHR*=0.99，95%CI (0.91-1.07)，*P*=0.80] *I*^2^=0%, Fixed effects model

**Figure S1**

1. Gender (female as the exposure factor)：[*aHR*=0.59, 95%*CI* (0.46-0.76), *P*<0.0001] *I*^2^=0%, Fixed effects model

**Figure S2**

1. History of alcohol：[*aHR*=0.70, 95%*CI* (0.50-0.98), *P*=0.039] *I^2^*=49%, Fixed effects model

**Figure S3**

1. History of smoking：[*aHR*=0.58, 95%*CI* (0.38-0.88), *P*=0.01]  *I*^2^=0%, Fixed effects model

**Figure S4**

1. Current smoker：[*aHR*=0.61, 95%*CI* (0.30-1.24), *P*=0.17]  *I*^2^=79.2%, Random effects model

**Figure S5**

1. TB treatment history：[*aHR*=0.94, 95%*CI* (0.83-1.09), *P*=0.46] *I*^2^=0%, Fixed effects model

**Figure S6**

1. History of SLD use：[*aHR*=0.64, 95%*CI* (0.47-0.87), *P*=0.004] *I*^2^=0%, Fixed effects model

**Figure S7**

1. BMI < 18.5kg/m^2^：[*aHR*=0.69, 95%*CI* (0.60-0.80), *P*<0.0001] *I*^2^=0%, Fixed effects model

**Figure S8**

1. Diabetes：[*aHR*=0.77, 95%*CI* (0.50-1.17), *P*=0.22] *I*^2^=79.1%, Random effects model

**Figure S9**

1. lung cavity：[*aHR*=0.70, 95%*CI* (0.52-0.94), *P*=0.016] *I*^2^=70.3%, Random effects model

**Figure S10**

1. HIV：[*aHR*=0.76, 95%*CI* (0.42-1.21), *P*=0.36] *I*^2^=0%, Fixed effects model

**Figure S11**

1. Sputum smear grading at baseline(positive)：[*aHR*=0.56, 95%*CI* (0.36-0.87), *P*=0.009] *I^2^*=50.3%, Random effects model

**Figure S12**

1. Sputum smear grading at baseline (grade^1+^): [*aHR*=0.87, 95%*CI* (0.77-0.99), *P*=0.043] *I*^2^=0%, Fixed effects model

**Figure S13**

1. Sputum smear grading at baseline (grade^2+^)：[*aHR*=0.81, 95%*CI* (0.69-0.95), *P*=0.009] *I*^2^=0%, Fixed effects model

**Figure S14**

1. Sputum smear grading at baseline (grade^3+^)：[*aHR*=0.71, 95%*CI* (0.61-0.84), *P*<0.0001] *I*^2^=0%, Fixed effects model

**Figure S15**

1. Resistance to ofloxacin：[*aHR*=0.67, 95%*CI* (0.43-1.04), *P*=0.07] *I*^2^=58.6%, Random effects model

**Figure S16**

1. Resistance to all five first lines drugs：[*aHR*=0.86, 95%*CI* (0.62-1.21), *P*=0.395] *I*^2^=0%, Fixed effects model

**Figure S17**
