# Supplementary material for: Time to sputum culture conversion and its associated factors among drug-resistant tuberculosis patients: a systematic review and meta-analysis
Source: BMC Infect Dis. 2024 Feb 7;24:169. doi: 10.1186/s12879-024-09009-5 (PMC10848338; doi:10.1186/s12879-024-09009-5)
Supplement: Supplementary file 2 — Additional file 2. [file 12879_2024_9009_MOESM2_ESM.docx]

**supplement(Included research)**

1. Yihunie Akalu T, Muchie KF, Alemu Gelaye K. Time to sputum culture conversion and its determinants among Multi-drug resistant Tuberculosis patients at public hospitals of the Amhara Regional State: A multicenter retrospective follow up study. PLoS One. 2018;13(6):e0199320.
2. Akinsola OJ, Yusuf OB, Ige OM, et al. Models for Predicting Time to Sputum Conversion Among Multi-Drug Resistant Tuberculosis Patients in Lagos, South-West Nigeria. Front Public Health. 2018;6:347.
3. Putri FA, Burhan E, Nawas A, et al. Body mass index predictive of sputum culture conversion among MDR-TB patients in Indonesia. Int J Tuberc Lung Dis. 2014 May;18(5):564-70.

[4] LU P. Analysis of the relationship between the sputum negative conversion time and the treatment outcome of MDR-TB and its influencing factors [D]. Nanjing Medical University, 2017.

[5] Li Q, LU M, WU LM, et al. Analysis of the influencing factors of sputum culture negative conversion in mdr-tb patients at the end of 6-month treatment. Chin J of Antituber, 2019, 41(08):869-875.

[6] Kurbatova EV, Gammino VM, Bayona J, Becerra MC, Danilovitz M, Falzon D, Gelmanova I, Keshavjee S, Leimane V, Mitnick CD, Quelapio MI, Riekstina V, Taylor A, Viiklepp P, Zignol M, Cegielski JP. Predictors of sputum culture conversion among patients treated for multidrug-resistant tuberculosis. Int J Tuberc Lung Dis. 2012 Oct;16(10):1335-43.

[7] Li Q, Lu M, Hsieh E, et al. Time to sputum culture conversion and its predictors among patients with multidrug-resistant tuberculosis in Hangzhou, China: A retrospective cohort study. Medicine (Baltimore). 2020;99(50):e23649.

[8] Liu Q, Lu P, Martinez L, et al. Factors affecting time to sputum culture conversion and treatment outcome of patients with multidrug-resistant tuberculosis in China. BMC Infect Dis. 2018;18(1):114.

[9] Magee MJ, Kempker RR, Kipiani M, et al. Diabetes mellitus, smoking status, and rate of sputum culture conversion in patients with multidrug-resistant tuberculosis: a cohort study from the country of Georgia. PLoS One. 2014;9(4):e94890.

[10] Ncha R, Variava E, Otwombe K, et al. Predictors of time to sputum culture conversion in multi-drug-resistant tuberculosis and extensively drug-resistant tuberculosis in patients at Tshepong-Klerksdorp Hospital. S Afr J Infect Dis. 2019;34(1):111.

[11] Mpagama SG, Heysell SK, Ndusilo ND, et al. Diagnosis and interim treatment outcomes from the first cohort of multidrug-resistant tuberculosis patients in Tanzania. PLoS One. 2013;8(5):e62034.

[12] Huerga H, Bastard M, Kamene M, et al. Outcomes from the first multidrug-resistant tuberculosis programme in Kenya. Int J Tuberc Lung Dis. 2017;21(3):314-319.

[13] Bade AB, Mega TA, Negera GZ. Malnutrition is Associated with Delayed Sputum Culture Conversion Among Patients Treated for MDR-TB. Infect Drug Resist. 2021;14:1659-1667.

[14] Brust JC, Lygizos M, Chaiyachati K, et al. Culture conversion among HIV co-infected multidrug-resistant tuberculosis patients in Tugela Ferry, South Africa. PLoS One. 2011;6(1):e15841.

[15] Magee MJ, Sun YV, Brust JCM, et al. Polymorphisms in the vitamin D receptor gene are associated with reduced rate of sputum culture conversion in multidrug-resistant tuberculosis patients in South Africa. PLoS One. 2017;12(7):e0180916.

[16] Brust JC, Berman AR, Zalta B, et al. Chest radiograph findings and time to culture conversion in patients with multidrug-resistant tuberculosis and HIV in Tugela Ferry, South Africa. PLoS One. 2013;8(9):e73975.

[17] Gadallah MA, Mokhtar A, Rady M, et al. Prognostic factors of treatment among patients with multidrug-resistant tuberculosis in Egypt. J Formos Med Assoc. 2016;115(11):997-1003. doi:10.1016/j.jfma.2015.10.002

[18] Zheng X, Davies Forsman L, Bao Z, et al. Drug exposure and susceptibility of second-line drugs correlate with treatment response in patients with multidrug-resistant tuberculosis: a multicentre prospective cohort study in China. Eur Respir J. 2022;59(3):2101925.

[19] Tierney DB, Franke MF, Becerra MC, et al. Time to culture conversion and regimen composition in multidrug-resistant tuberculosis treatment. PLoS One. 2014;9(9):e108035.

[20] Lee M, Han J, Kim YR, et al. Multidrug-resistant tuberculosis in South Korea: a retrospective analysis of national registry data in 2011-2015. Int J Tuberc Lung Dis. 2019;23(7):850-857.

[21] Javaid A, Ahmad N, Afridi AK, et al. Validity of Time to Sputum Culture Conversion to Predict Cure in Patients with Multidrug-Resistant Tuberculosis: A Retrospective Single-Center Study. Am J Trop Med Hyg. 2018;98(6):1629-1636.

[22] Hafkin J, Modongo C, Newcomb C, et al. Impact of the human immunodeficiency virus on early multidrug-resistant tuberculosis treatment outcomes in Botswana. Int J Tuberc Lung Dis. 2013;17(3):348-353.

[23] Ghimire S, Karki S, Maharjan B, et al. Treatment outcomes of patients with MDR-TB in Nepal on a current programmatic standardised regimen: retrospective single-centre study. BMJ Open Respir Res. 2020;7(1):e000606.

[24] Diktanas S, Korotych O, Sereda Y, et al. Factors associated with time to sputum culture conversion of rifampicin-resistant tuberculosis patients in Klaipeda, Lithuania in 2016-2019: a cohort study. Monaldi Arch Chest Dis. 2021;91(1):10.4081/monaldi.2021.1675.

[25] Meshesha MD. Predictors of sputum culture conversion time among MDR/RR TB patients on treatment in a low-income setting. PLoS One. 2022;17(11):e0277642.

[26] Tekalegn Y, Woldeyohannes D, Assefa T, et al. Predictors of Time to Sputum Culture Conversion Among Drug-Resistant Tuberculosis Patients in Oromia Region Hospitals, Ethiopia. Infect Drug Resist. 2020;13:2547-2556.

[27] Velayutham B, Nair D, Kannan T, et al. Factors associated with sputum culture conversion in multidrug-resistant pulmonary tuberculosis. Int J Tuberc Lung Dis. 2016;20(12):1671-1676.

[28] Diallo A, Diallo BD, Camara LM, et al. Different profiles of body mass index variation among patients with multidrug-resistant tuberculosis: a retrospective cohort study. BMC Infect Dis. 2020;20(1):315.

[29] Reimann M, Schaub D, Kalsdorf B, et al. Cigarette smoking and culture conversion in patients with susceptible and M/XDR-TB. Int J Tuberc Lung Dis. 2019;23(1):93-98.

[30] Shibabaw A, Gelaw B, Wang SH, et al. Time to sputum smear and culture conversions in multidrug resistant tuberculosis at University of Gondar Hospital, Northwest Ethiopia. PLoS One. 2018;13(6):e0198080.

[31] Ding CH, Xiong Y, Wang Q, et al. Early efficacy and safety of bedaquinoline-containing regimen in the treatment of multi-drug resistant pulmonary tuberculosis. Chin J of Antituber, 2021, 43(09):893-898.

[32] Htun YM, Khaing TMM, Aung NM, et al. Delay in treatment initiation and treatment outcomes among adult patients with multidrug-resistant tuberculosis at Yangon Regional Tuberculosis Centre, Myanmar: A retrospective study. PLoS One. 2018;13(12):e0209932.

[33] Shi ZY, Wu GH, Huang T,et al. 24-week, single-group, observational study of a bedaquinoline-containing regimen in the treatment of multidrug-resistant Extensively drug-resistant tuberculosis disease. Chin J of Antituber, 2021,43(05):487-494.

[34] Kim CT, Kim TO, Shin HJ, et al. Bedaquiline and delamanid for the treatment of multidrug-resistant tuberculosis: a multicentre cohort study in Korea. Eur Respir J. 2018;51(3):1702467.

[35] Abubakar M, Ahmad N, Atif M, et al. Prognostic accuracy of time to sputum culture conversion in predicting cure in extensively drug-resistant tuberculosis patients: a multicentre retrospective observational study. BMC Infect Dis. 2022;22(1):204.

[36] Salindri AD, Kipiani M, Kempker RR, et al. Diabetes Reduces the Rate of Sputum Culture Conversion in Patients With Newly Diagnosed Multidrug-Resistant Tuberculosis. Open Forum Infect Dis. 2016;3(3):ofw126.

[37] Wu GL, Gao JT, Chen XH, et al. Short-term efficacy and safety of a bedaquinoline-containing regimen in the treatment of multidrug-resistant Extensively drug-resistant tuberculosis disease. Chin J of Antituber, 2021, 43(09):899-904.

[38] Pei Y, Gao JT，Huang YH，et al. A 24-week analysis of the efficacy of bedaquinoline-containing regimen in the treatment of 44 patients with sputum-positive multi-drug resistant/extensively drug resistant pulmonary tuberculosis. Chin J of Antituber, 2021, 43(11):1139-1145.

[39] KIM J, KWAK N, LEE H Y, et al. Effect of drug resistance on negative conversion of sputum culture in patients with pulmonary tuberculosis[J]. International Journal of Infectious Diseases, 2016, 42:64-68.

[40] Shi L, Gao J, Gao M, et al. Interim Effectiveness and Safety Comparison of Bedaquiline-Containing Regimens for Treatment of Diabetic Versus Non-Diabetic MDR/XDR-TB Patients in China: A Multicenter Retrospective Cohort Study. Infect Dis Ther. 2021;10(1):457-470.

[41] Rodriguez M, Monedero I, Caminero JA, et al. Successful management of multidrug-resistant tuberculosis under programme conditions in the Dominican Republic. Int J Tuberc Lung Dis. 2013;17(4):520-525.

[42] Parmar MM, Sachdeva KS, Dewan PK, et al. Unacceptable treatment outcomes and associated factors among India's initial cohorts of multidrug-resistant tuberculosis (MDR-TB) patients under the revised national TB control programme (2007-2011): Evidence leading to policy enhancement. PLoS One. 2018;13(4):e0193903.

[43] Heyckendorf J, van Leth F, Avsar K, et al. Treatment responses in multidrug-resistant tuberculosis in Germany. Int J Tuberc Lung Dis. 2018;22(4):399-406. doi:10.5588/ijtld.17.0741

[44] Gao M, Gao J, Xie L, et al. Early outcome and safety of bedaquiline-containing regimens for treatment of MDR- and XDR-TB in China: a multicentre study. Clin Microbiol Infect. 2021;27(4):597-602.

[45] Borisov SE, Dheda K, Enwerem M, et al. Effectiveness and safety of bedaquiline-containing regimens in the treatment of MDR- and XDR-TB: a multicentre study. Eur Respir J. 2017;49(5):1700387.
